# Modified anterior approach preserving Retzius space versus standard anterior approach robot-assisted radical prostatectomy: A matched-pair analysis

**DOI:** 10.3389/fonc.2023.1108202

**Published:** 2023-02-02

**Authors:** Hui Li, Chao Yang, Zhonghong Liao, Kaihong Wang, Yida Zhang, Runfu Cao

**Affiliations:** Department of Urology, The First Affiliated Hospital of Nanchang University, Nanchang, China

**Keywords:** prostate cancer, prostatectomy, robotic-assisted surgery, Retzius sparing, modified anterior approach

## Abstract

**Objective:**

To compare our initial perioperative and postoperative outcomes of the modified anterior approach (MA) with Retzius space preservation robot-assisted radical prostatectomy (RARP) with the standard anterior approach (SA) RARP.

**Materials and methods:**

A retrospective analysis was performed on 116 patients with RARP completed by the same surgeon between September 2019 and March 2022. They were divided into SA-RARP group (77 cases) and MA-RARP group (39 cases). Propensity score matching was performed using eight preoperative variables, including age, BMI, preoperative PSA, biopsy Gleason score, prostate volume, D’Amico risk classification, SHIM, and clinical T stage. Functional outcome was assessed by urine pad count and SHIM after surgery, and oncological outcome was assessed by statistics of postoperative pathological findings as well as follow-up postoperative PSA. The median follow-up was 13 months and 17 months for MA-RARP and SA-RARP groups respectively.

**Results:**

Propensity score matching was performed 1:1, and baseline data were comparable between the two groups after matching. Comparison of postoperative data: MA-RARP group had less mean EBL than SA-RARP group (200 vs 150 ml, p = 0.033). PSM did not differ between groups (p = 1). In terms of urinary control recovery, the MA-RARP group showed significant advantages in urinary control recovery at 24 h, 2 weeks, 1 month and 3 months after catheter removal, respectively (48.6% vs 5.7%, p < 0.001; 80% vs 22.9%, p < 0.001; 94.3% vs 51.4%, p < 0.001; 100% vs 74.3%, p = 0.002). This advantage gradually disappeared 6 months or more after surgery. The median time to recovery of sexual function was shorter in the MA-RARP group (165 vs 255 d, p = 0.001).

**Conclusion:**

MA-RARP is safe and reliable, and can achieve better early urinary control function and sexual function recovery while achieving the primary tumor control goal.

## 1 Introduction

Globally, the incidence of Prostate Cancer is second only to lung cancer, ranking the second among the most common malignant tumors in men (1).Radical Prostatectomy (RP) is an effective surgical method for the treatment of prostate cancer, and is the first choice of treatment for localized prostate cancer. In recent years, due to its unique 3D vision and flexible robotic arm operation, surgical robot has been used by more and more surgeons as the preferred method for radical prostatectomy. However, decreased urinary control and loss of sexual function are the main factors affecting the quality of life of patients. How to achieve the “long-term tumor control, retention of urinary control function, retention of erectile function, no surgical complications and negative specimen margin” of the five consecutive operations, is the urological surgeons have been pursuing to achieve the goal. To achieve this goal, various robot-assisted radical prostatectomy (RARP) approaches have been explored by expert teams around the world, including anterior, posterior, transvesical, lateral, and perineal approaches (2, 3).

The anterior approach is the most widely accepted surgical approach by urologists, and it is also the classic standard surgical approach. The anterior approach is mainly represented by the “Veil of Aphrodite “ technique and “Vattikuti” prostatectomy *via* retropubic space approach (4, 5). The modified anterior approach preserves the Retzius space for robot-assisted laparoscopic radical prostatectomy, which is modified on the basis of the standard anterior approach, using the no-clip technique and bladder neck preservation technique, in order to achieve the goal of tumor control and achieve better functional preservation. Therefore, the purpose of this study was to evaluate the differences in efficacy and safety between the modified anterior approach robot-assisted radical prostatectomy (MA-RARP) and the standard anterior approach robot-assisted radical prostatectomy (SA-RARP).

## 2 Patients and methods

### 2.1 Patient selection

The clinical data of RARP patients in the Department of Urology, the First Affiliated Hospital of Nanchang University from September 2019 to March 2022 were retrospectively analyzed. According to different surgical methods, the patients were divided into MA-RARP group and SA-RARP group. The operation was performed by an experienced urological surgeon through the abdominal approach in both groups (number of operations >200). Among them, 39 patients underwent modified anterior approach to preserve Retzius space. There were 77 cases in the standard anterior approach group. Patients who had previously undergone surgery for transurethral resection of the prostate (TURP) or neoadjuvant androgen deprivation therapy (ADT) were excluded from the group.

### 2.2 Surgical technique

The procedure was performed using the standard Da Vinci Si surgical robot. Patients were generally anesthetized in a head-down, foot-up, 15˚ flat position and performed in a 5-tubing approach. A pneumoperitoneum was established with a pneumoperitoneum needle 2 cm above the umbilicus, and the abdominal pressure was maintained at 12-14 mmHg. A 2-cm incision was made longitudinally at the location of the pneumoperitoneum needle, and a 12-mm trocar was inserted and fixed with sutures, and the laparoscope was introduced. Under laparoscopic surveillance, two 8 mm trocar holes were placed at 8 cm from the umbilicus at the level of one finger below the umbilicus on both sides, and the right side was connected to the robotic arm No. 1 monopolar electric scissors, and the left side was connected to the robotic arm No. 2 maryland bipolar grasping forceps, and then a hole was made in 8 cm below the left side hole to place the 8 mm trocar to connect to the robotic arm No. 3 human prograp grasping forceps, a hole was made 8 cm down the right side of the hole and a 5mm trocar was placed for suction. Enlarged pelvic lymph node dissection was performed for cases with >5% probability of lymph node metastasis. The standard anterior approach was done in the “Vattikuti” technique *via* the retzius space as proposed by Menon’s team in 2003 (4). Modified anterior approach procedure:

#### 2.2.1 Only an inverted U shape was used to free the anterior surface of the bladder to establish the operating plane

A bilateral incision was made over the peritoneal covering lateral to the umbilical ligament, preserving the midline umbilical ureteric ligament, exposing the bladder and not revealing the prostate contour after excision of the overlying adipose tissue close to the bladder muscle to avoid excessive freeing of the bladder neck and prostate flanks.

#### 2.2.2 Delicate separation of the bladder neck with maximum preservation of the bladder sphincter

**Figure 1:**
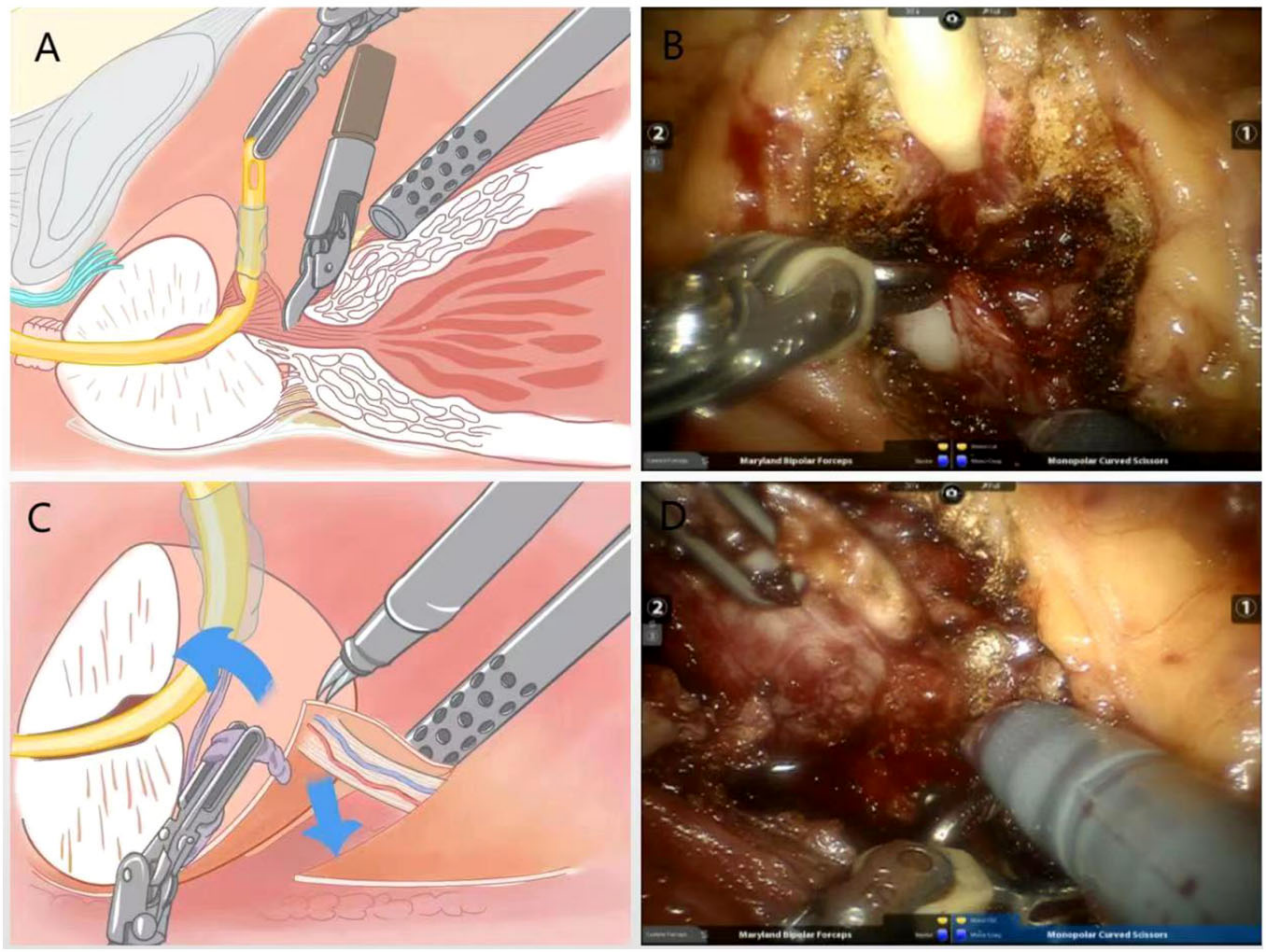
**(A)** Schematic diagram of bladder neck preserving surgery; **(B)** Practical diagram; **(C)** Schematic diagram of the surgical treatment of the right vascular tip of the prostate using the no-clips technique; **(D)** Practical diagram.

Following the surgical technique of preserving the bladder neck proposed by Freire et al. (6) the bladder-prostate junction was identified by pulling the ureter with an assistant. The prostate-covered bladder sphincter fibers were exposed using a combination of sharp and blunt separation, and then the bladder neck is dissociated to ultimately achieve preservation of the funnel-shaped bladder neck (**Figures 1A, B**).

#### 2.2.3 Separation of the dorsal aspect of the prostate and non-thermal technique to dissect the vas deferens and seminal vesicles

After dissection of the bladder neck, the vas deferens and the seminal vesicles are searched for and then dissected using a non-thermal technique. Denonvilliers’ fascia was observed by pulling the seminal vesicles and vas deferens ventrally. Near the seminal vesicle-prostate junction, there is fusion of denonvilliers’ fascia with the prostate capsule. The denonvilliers’ fascia is incised in the midline between the seminal vesicle and the rectum, which avoids damaging the prostate capsule. The anatomical plane of the prostate capsule is then separated as close as possible to the tip and laterally (**Figures 1C, D**).

#### 2.2.4 Separation of the lateral aspect of the prostate with full preservation of the neurovascular bundle

**Figure 2:**
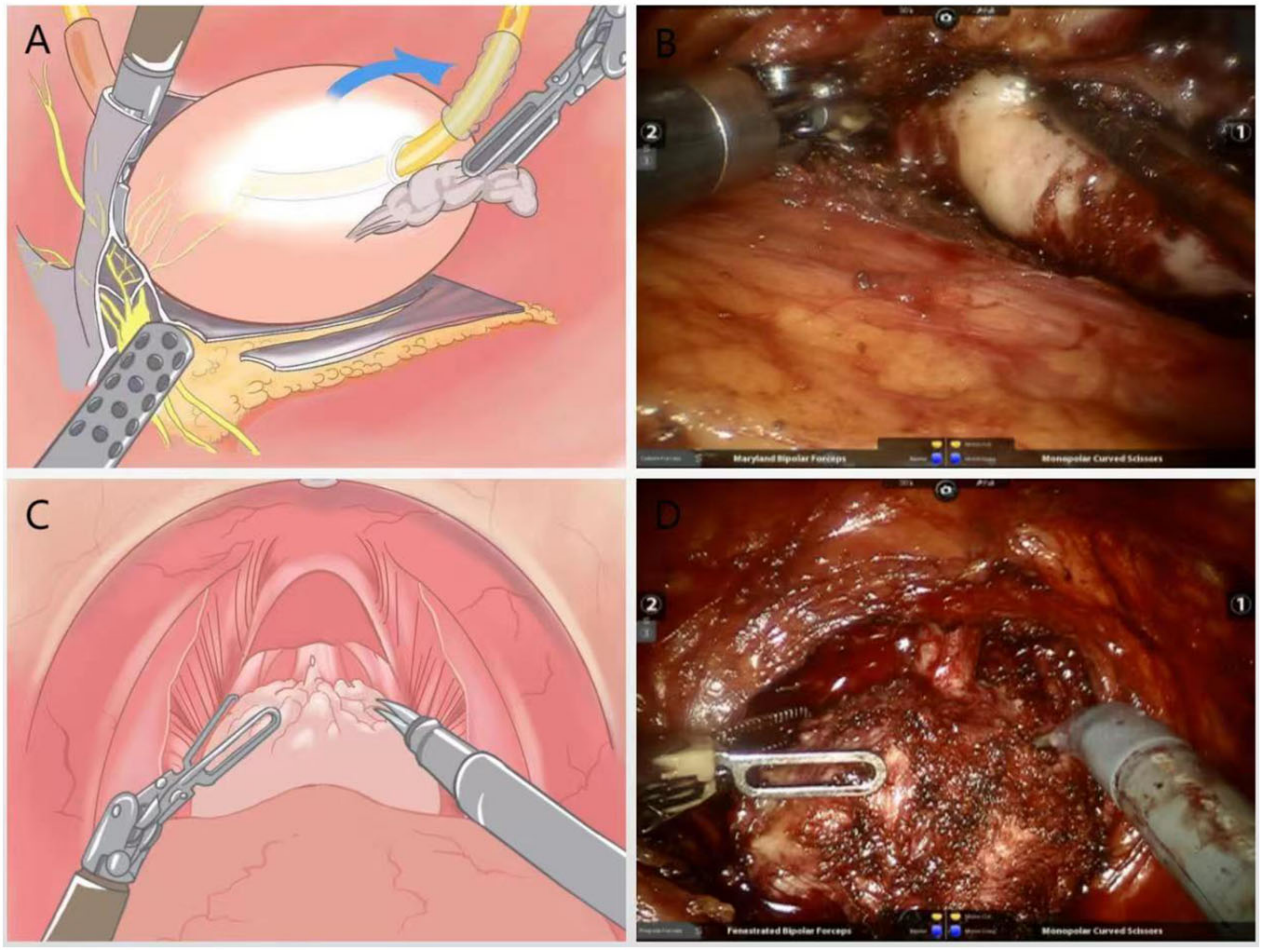
**(A)** Schematic diagram of the lateral dissection of the prostate with intrafascial technique to preserve NVB; **(B)** Practical diagram; **(C)** Schematic diagram of surgery to strip the urethral sphincter and maximize preservation of the membranous urethra; **(D)** Practical diagram.

A combination of blunt and sharp separation was used to free the apical and lateral aspect. The gap was separated at 4-5 points on the lateral side of the prostate against the prostatic capsule, and the visceral endopelvic fascia was pushed through the dirty layer with electric scissors to reveal the right vascular tract of the prostate.The vascular pedicle on the right side of the prostate was severed with a cold knife without the use of a vascular clamp. For a small amount of bleeding, accurate hemostasis was performed with low-energy electrocoagulation, and attention was paid to avoiding “transection” of NVB. The left vascular tip was treated in the same way (**Figures 2A, B**).

#### 2.2.5 Separation of the ventral and apical portions of the prostate without freeing and without suturing the dorsal venous complex

Under the DVC, the anterior surface of the prostate was separated anteriorly against the capsule until the apex, without opening the intrapelvic fascia to avoid dissociation and ligation of the DVC.The blunt and sharp combination method was used to carefully dissociate the tip of the prostate, identifying the junction between the membranous urethra and the prostate. The striated urethra sphincter was bluntly separated at the tip, and the part of the urethra covered by the prost gland tissue was removed, and the membranous urethra was preserved to the maximum extent (**Figures 2C, D**).

#### 2.2.6 Complete resection of prostate and accurate anastomosis of bladder and urethra

**Figure 3:**
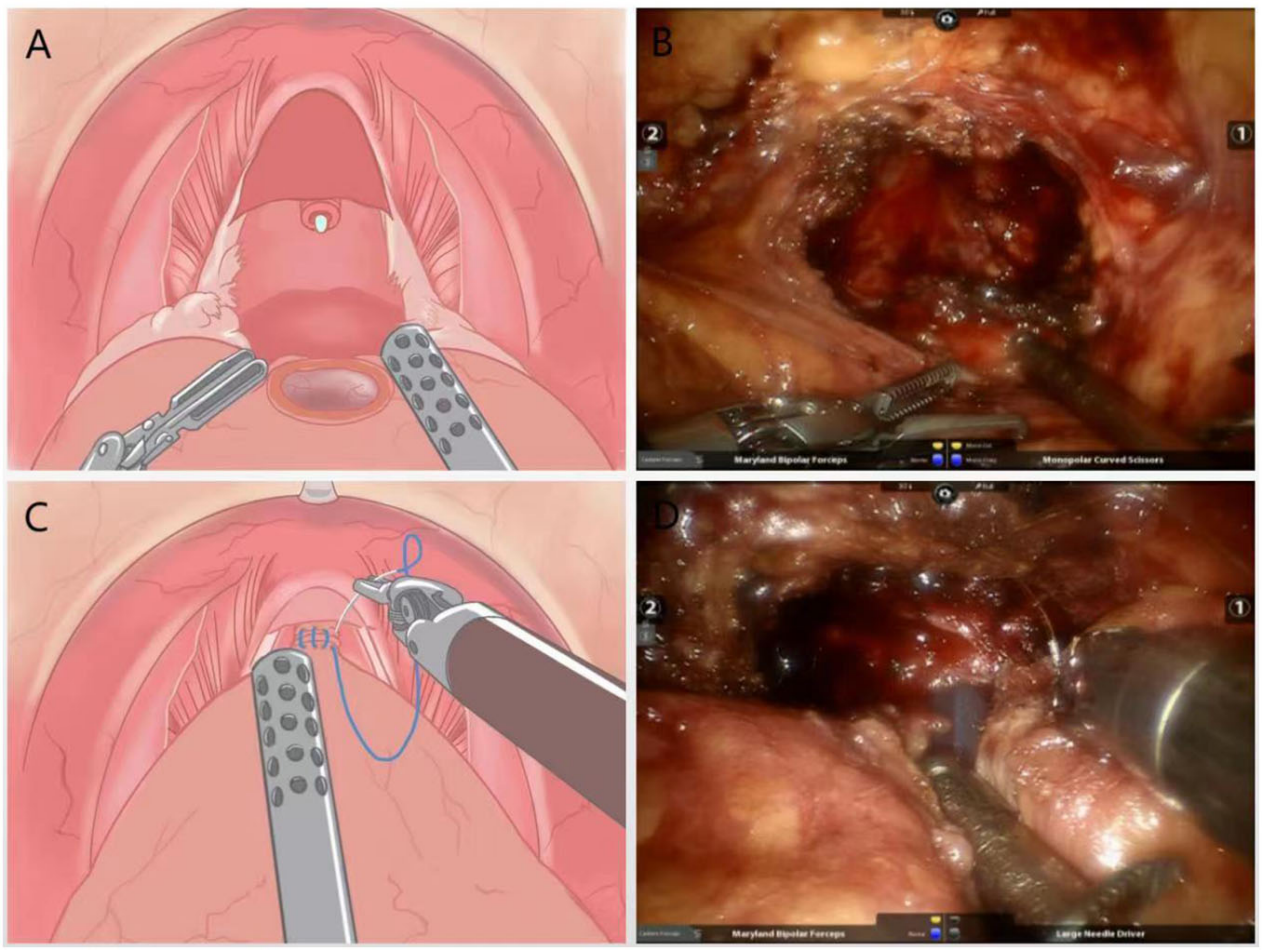
**(A)** Schematic diagram of the surgery to remove the prostate and preserve the “hood” structure around the gland; **(B)** Practical diagram; **(C)** Schematic diagram of anatomical reduction surgery with double stitches to close the urethra and bladder neck; **(D)** Practical diagram.

The urethra was dissected and the prostate is removed, preserving the “hood” structure around the gland (**Figures 3A, B**). A precise anastomosis between the urethral section and the bladder neck is performed, and for patients with a large middle lobe prostate, a “racket” suture was used for double-layer bladder neck reconstruction followed by anastomosis and final suturing of the peritoneum (**Figures 3C, D**).

### 2.3 Evaluated variables

For all patients included in the study, we collected data regarding age, body mass index (BMI), prostate specific antigen (PSA), puncture biopsy Gleason score, number of positive puncture stitches and percentage of positives, and assessed patients’ D’Amico risk classification. All patients underwent preoperative multiparametric magnetic resonance imaging (MRI) or computed tomography (CT), as well as whole-body bone scan imaging to assess clinical TNM stage, and a statistical survey of sexually active patientssexual health index for men (SHIM). Data on operative time, estimated blood loss (EBL), whether blood was transfused. The *ad hoc* group following the EAU guidelines proposed standardized collection procedures and collected statistics of perioperative complications in all patients according to the Clavien-Dindo classification (7). Postoperative pathological data were collected from patients, including Gleason score, extraperitoneal extension, seminal vesicle invasion, positive surgical margins (PSM), and pathological TNM stage.

Patients were treated perioperatively in the usual way at our institution. The Foley catheter was removed 7 to 8 days after surgery in the MA-RARP group; the Foley catheter was removed 8 to 10 days after surgery in the SA-RARP group. Patients with stage ≤ pT2, Gleason score ≤ 7 and negative incision margins were not ADT. follow-up data on urinary control at 24 hours, 2 weeks, 1 month, 3 months and 6 months after removal of the urinary catheter were counted (normal criteria for urinary control was 0-1 urinary pad use per day) (8); SHIM questionnaire was administered to patients with normal preoperative sexual function at 3 and 6 months after surgery (normal criteria for sexual function was SHIM ≥ 17); PSA data follow-up was performed at 1 week, 3 months, and 6 months postoperatively (biochemical recurrence (BCR) defined as two consecutive assayed academic PSA > 0.2 ng/ml (9)). Analysis was performed to summarize the recovery of urinary control and erectile function as well as oncological prognosis.

### 2.4 Statistical analysis

A PM analysis was performed to eliminate the impact of significant differences in preoperative clinical characteristics between the SA-RARP and MA-RARP groups. All preoperative features were taken into account to estimate the propensity score *via* applying non-parsimonious and multivariate logistic regression. Finally, 35 patients in the SA-RARP group were successfully matched to patients treated with MA-RARP in a 1:1 ratio in accordance to the nearest neighbor matching method within the matching strategy(matching tolerance =0.03). The covariate differences were compared before and after matching to delineate the improved balance between the procedure arms after PM.

Matching resulted in 35 cases included in each group, for a total of 70 cases in our study. Descriptive statistics were performed for all variables. Categorical variables were expressed as number of cases and percentages, and differences between the two groups were assessed using chi-square tests and Fisher’s exact tests, as appropriate. And continuous variables were expressed using mean and standard deviation (SD) or median and interquartile range (IQR), as appropriate, and continuous variables were analyzed using Student t-test or the MannWhitney U test, as appropriate. All statistical analyses were done using IBM SPSS Statistics version 26.0. All tests were two-sided, with a significance set at p<0.05.

## 3 Results

### 3.1 Study population

Among the 39 patients in the MA-RARP group, 35 patients were finally included in the MA-RARP group because 2 patients had a history of TURP surgery and 2 patients underwent ADT after biopsy diagnosis of prostate cancer. After 1:1 matching of patients in the SA-RARP group, age, BMI, presurgical PSA, biopsy Gleason score, prostate volume, D ‘Amico risk classification, SHIM, and clinical T stage were comparable (**Table 1**). Before operation, 21(60%) patients in MA-RARP group had SHIM score≥17, and 19(54.3%) patients in SA-RARP group had SHIM score≥17. All patients could achieve urinary flow control before operation.

**Table 1:** Post-matching baseline characteristics of 35 patients who underwent SA-RARP and 35 patients who underwent MA-RARP.

Variable	MA-RARP (N=35)	Before propensity score matching		After propensity score matching	
		SA-RARP (N=77)	p value	SA-RARP (N=35)	p value
Mean ± SD age (years)	66.9 ± 6.0	68.5 ± 7.2	0.248	66.4 ± 6.0	0.705
Mean ± SD BMI (kg/m2)	24.6 ± 2.8	24.2 ± 2.6	0.386	24.4 ± 2.9	0.678
Median preoperative tPSA, ng/mL (IQR)	10.7 (7.3, 14.8)	17.5 (10.0, 23.9)	<0.001	12.0 (7.4, 17.8	0.643
Biopsy Gleason score, N (%)			0.001		0.422
≤6	10.0 (28.6%)	11.0 (14.3%)		9.0 (25.7%)	
7 (3 + 4)	11.0 (31.4%)	11.0 (14.3%)		8.0 (22.9%)	
7 (4 + 3)	8.0 (22.9%)	10.0 (13%)		6.0 (17.1%)	
≥8	6.0 (17.1%)	45.0 (58.4%)		12.0 (34.3%)	
D’Amico risk, N (%)			0.030		0.767
Low	7.0 (20.0%)	8.0 (10.4%)		8.0 (22.9%)	
Intermediate	16.0 (45.7%)	22.0 (28.6%)		13.0 (37.1%)	
High	12.0 (34.3%)	47.0 (61.0%)		14.0 (40.0%)	
Median prostate volume, cc (IQR)	27.0 (21.8, 35.8)	32.5 (24.9, 44.8)	0.035	32.1 (24.5, 42.2)	0.154
Clinical T stage, N (%)			0.066		0.552
T1	5.0 (14.3%)	5.0 (6.5%)		5.0 (14.3%)	
T2	26.0 (74.3%)	49.0 (63.6%)		29.0 (82.9%)	
≥T3	4.0 (11.4%)	23.0 (29.9%)		1.0 (2.9%)	
Preoperative SHIM, N (%)			0.241		0.629
SHIM score≥17	21.0 (60.0%)	37.0 (48.1%)		19.0 (54.3%)	
SHIM score<17	14.0 (40.0%)	40.0 (51.9%)		16.0 (45.7%)	

### 3.2 Perioperative outcomes

The mean surgical EBL was less in the MA-RARP group than in the SA-RARP group (200 vs 150 ml, p=0.033). Postoperative hospital stay (7 vs 6 d, p=0.032) and catheter removal time (9 vs 7 d, p<0.001) were shorter in the MA-RARP group. The incidence of postoperative complications was not statistically significant between the two groups (**Table 2**). One patient in the SA-RARP group developed grade IV complications of cerebral artery stenosis and occlusion and was transferred to the Intensive care unit(ICU)for further treatment. Among the other 11 grade I/II complications, 4 patients received blood transfusion due to postoperative hemoglobin decrease; Two patients had anastomotic leakage and were treated by prolonged catheter removal; Two patients had bladder urethral stricture and were treated with urinary tract dilation. Two patients had asymptomatic urinary tract infections and were treated with oral antibiotics; One patient developed lymphatic leakage and was treated by prolonged placement of a drainage tube.The mean operation time of MA-RARP group was longer than that of SA-RARP group, but there was no significant difference between the two groups. There was no significant difference in intraoperative and postoperative blood transfusion between the two groups. In general, all 70 patients in the two groups successfully completed surgery, with no conversion to open surgery and no readmission due to postoperative complications.

**Table 2:** Procedure specifific complications, stratifified by Clavien-Dindo classifification in 35 men treated with MA-RARP and 35 treated with SA-RARP.

Grades	Procedurespecifific	SA-RARP(N=35)	MA-RARP(N=35)	pvalue
I	Asymptomaticurinarytractinfection	1(2.9%)	1(2.9%)	1.000
	Vesico-urethralanastomosisleakingurine	1(2.9%)	1(2.9%)	
	Urethralstricture	1(2.9%)	1(2.9%)	
	Lymphaticleak	1(2.9%)	0	
II	Bloodtransfusion	3(8.6%)	1(2.9%)	0.614
III	NA	0	0	NA
IV	Cerebralarterystenosisandoccludedcerebralinfarction	1(2.9%)	0	1.000
V	NA	0	0	NA
Total, N(%)	NA	8(22.9%)	4(11.4%)	0.205

NA, Data is lost or unavailable

### 3.3 Oncology outcomes

There was no significant difference in PSM between the two groups (p=1). Gleason score and pathological TNM stage were not significantly different between the two groups (p = 0.103, 0.691, respectively). However, in the MA-RARP group, all the 4 cases with positive surgical margins were at T3 stage and at high risk according to D ‘Amico risk classification. Among them, 3 cases had positive sites at the tip, and 1 case had positive sites at the tip and bottom. In terms of pelvic lymph node dissection, there was no statistical significance between the two groups: 17 patients in the MA-RARP group and 19 patients in the SA-RARP group underwent enlarged pelvic lymph node dissection, and postoperative pathology indicated that one patient in the two groups had positive lymph nodes, respectively. The PSA data of 66, 58 and 55 patients were collected by outpatient review or telephone follow-up at 1 week, 3 months and 6 months after operation, and the results showed that there was no significant difference in PSA values between the two groups (p = 0.308, 0.778 and 0.840, respectively). The number of patients with BCR was comparable between the two groups during 7-36 months of follow-up (1 vs 2, p = 1) (**Table 3**).

**Table 3:** Post-matching perioperative and postoperative outcomes of 35 patients who underwent SA-RARP vs 35 patients who underwent MA-RARP.

	SA-RARP (N=35)	MA-RARP (N=35)	p value
Median operative time, mins (IQR)	160.0 (140.0, 180.0)	175.0 (140.0, 210.0)	0.305
Median estimated blood loss, ml (IQR)	200.0 (100.0, 200.0)	150.0 (150.0, 200.0)	0.033
Transfusion, N (%)	3.0 (8.6%)	1.0 (2.9%)	0.614
Median length of stay, days (IQR)	7.0 (5.0, 9.0)	6.0 (5.0, 7.0)	0.032
Median catheter removal, days (IQR)	9.0 (9.0, 9.0)	7.0 (7.0, 7.0)	<0.001
Pathological Gleason score, N (%)			0.103
6	6.0 (17.1%)	3.0 (8.6%)	
7 (3 + 4)	6.0 (17.1%)	15.0 (42.9%)	
7 (4 + 3)	9.0 (25.7%)	9.0 (25.7%)	
8	12.0 (34.3%)	8.0 (22.9%)	
9 (5 + 4)	2.0 (5.7%)	0.0 (100%)	
Pathologic stage, N (%)			0.403
T2	28.0 (80.0%)	25.0 (71.4%)	
T3	7.0 (20.0%)	10.0 (28.6%)	
ePLND, N (%)	19.0 (54.3%)	17.0 (48.6%)	0.632
PSM (%).	3.0 (8.6%)	4.0 (11.4%)	1.000
Immediate continence, N (%)	2.0 (5.7%)	17.0 (48.6%)	<0.001
2week continence, N (%)	8.0 (22.9%)	28.0 (80.0%)	<0.001
1 month continence, N (%)	18.0 (51.4%)	33.0 (94.3%)	<0.001
3 month continence, N (%)	26.0 (74.3%)	35.0 (100%)	0.002
6month continence, N (%)	31.0 (88.6%)	35.0 (100%)	0.114
SHIM score at 3 months, N (%)			0.089
<12	13.0 (68.4%)	7.0 (33.3%)	
12–16	3.0 (15.8%)	9.0 (42.9%)	
≥17	3.0 (15.8%)	5.0 (23.8%)	
SHIM score at 6 months, N (%)			0.127
<12	7.0 (36.8%)	2.0 (9.5%)	
12–16	6.0 (31.6%)	7.0 (33.3%)	
≥17	6.0 (31.6%)	12.0 (57.1%)	
Median postoperative tPSA, ng/ml (IQR)			
1 week (N=66)	2.15 (1.855, 2.475)	1.88 (1.57, 2.60)	0.308
3 months (N=58)	0.06 (0.02, 0.08)	0.05 (0.025, 0.09)	0.778
6 months (N=55)	0.01 (0.01, 0.02)	0.01 (0.01, 0.02)	0.840
BCR, N (%)	2 (5.7%)	1 (2.9%)	1.000

### 3.4 Functional outcomes

For recovery of urinary control, 24 h after catheter removal in the MA-RARP group (48.6% vs 5.7%, p<0.001), 2 weeks (80% vs 22.9%, p<0.001), 1 month (94.3% vs 51.4%, p<0.001), 3 months (100% vs 74.3%, p=0.002) had a significant advantage in urinary continence, which gradually disappeared at 6 months or longer after surgery (**Table 3**). In terms of sexual function, there were more cases in the MA-RARP group in the SHIM 12-16 range at 3 months after operation and in the SHIM 17-25 range at 6 months after operation, and the median time of sexual function recovery (SHIM≥17) was shorter in the MA-RARP group than in the SA-RARP group (165 vs 255 d, P = 0.001).

## 4 Discussion

Modified anterior approach preserving the retzius space RARP is modified from the standard anterior approach technique using a non-vascular clip technique. Various types of vessel clips are usually used to reduce bleeding on the cutting surface and maintain clear visual field when the lateral vascular pedicle of the prostate is dissociated by standard anterior approach. For a small amount of bleeding in the modified anterior approach, the use of low-energy electrocoagulation for precise hemostasis can avoid the symptoms of lower urinary tract irritation, perineal pain, hematuria and urination obstruction caused by vascular clamps around the bladder and urethra. At the same time, the non-vascular clip technique is helpful for the early recovery of sexual function. Another major improvement is the use of bladder neck preservation technique: standard anterior approach is usually performed by pulling the catheter to confirm the position of the bladder neck and disconnecting it directly; After the position of the bladder neck was confirmed by the modified anterior approach technique, the bladder sphincter fibers covered by the prostate were exposed by the combination of sharp and blunt separation, and then the bladder neck was severed to preserve the funnel shape of the bladder neck, which was helpful for the recovery of early postoperative urinary control. In addition, the modified anterior approach was performed as a complete intrafascial resection. The intrapelvic fascia is not opened, and the DVC is not dissected or sutured. Ganzer et al. demonstrated that at the tip of the prostate and 5 mm distal to the tip of the prostate, the DVC overlapped laterally with 37% and 30% of the urethral sphincter, respectively. In the case of lateral DVC ligation, most of the sphincter tissues may be ligated together and render them non-functional (10). Therefore, non-free and suture-free DVC can preserve the function of urethral sphincter to the greatest extent, and promote the recovery of urinary control and sexual function after surgery.

The prostatic plexus, intrapelvic fascia, Pubic prostatic ligament, detrusor apron and other periurethral support structures were preserved by the modified anterior approach to preserve the Retzius space robot-assisted laparoscopic radical profascial prostatectomy. Compared with SA-RARP, MA-RARP could shorten the postoperative hospital stay (6 vs 7 d, p=0.032) and accelerate the time of catheter removal (7 vs 9 d, p < 0.001), and MA-RARP significantly reduced EBL (150 vs 200 ml, p=0.033). There was a more advantage in urinary flow control in the first 3 months after surgery (100% vs 74.3%, p=0.002), but this advantage gradually disappeared with the extension of postoperative time. This is consistent with the conclusions of Albisinni et al. in their systematic review of the efficacy of the anterior approach versus robot-assisted radical prostatectomy with Retzius retained (11). In terms of sexual function, the MA-RARP technique significantly reduced the recovery time of sexual function (median 165 vs. 255 d, p=0.001). Compared with the “Hood” technique carried out by Wagaskar et al. (the urinary control at 2 weeks, 1 month and 3 months after catheter removal were 36%, 83% and 91%, respectively), the early urinary control recovery was faster (12). Compared with bladder neck preservation and additional anterior urethral fixation (65.6% urinary control at 4 months after operation), early urinary control recovery was faster (6).

It is well known that preservation of tissue structure during surgery results in better postoperative urinary flow control and sexual function recovery than structural reconstruction. The modified anterior approach retained Retzius space technique and Student et al. performed advanced reconstruction of vesico-urethral support (urine control rate was 62.5% at 4 weeks after surgery) (13), Porpiglia et al. The data of postoperative recovery of urinary incontinence in 1000 patients using total anatomical reconstruction technique were compared (the rate of urinary control was 79.66% at 4 weeks and 90.48% at 12 weeks) with the early recovery of urinary control (14, 15). The operation time of MA-RARP is longer than that of SA-RARP because more structures were preserved during operation. At the same time, we preserved the membranous urethra to the maximum extent during the operation, so that the positive surgical margin was mostly located in the tip. Li et al. demonstrated that preoperative pelvic MRI to determine the location of cancer foci was an independent predictor of positive surgical margin status after Retzius space RARP (16). To solve the problem of positive resection margin, we suggest to improve the relevant examination before surgery, especially pelvic MRI examination before surgery, to fully evaluate the tumor stage and the location of cancer foci. At the same time, prostate volume is also a potential factor affecting the postoperative functional outcome of patients. However, according to the study conducted by Galfano et al. on the influence of prostate volume on the function of retzius RARP and the outcome of oncology, as reported: retzius sparing RARP is feasible for prostate patients of any size, with similar oncologic and functional results (17). However, we recommend that surgeons who perform this technique at an early stage choose to use MA-RARP in patients with early limitation and small prostate volume.

A modified anterior approach preserving the Retzius space robot-assisted laparoscopic radical intrafascial resection of prostate cancer has shown good early urinary control and recovery of sexual function, probably due to the intraoperative preservation of the structures associated with the Retzius space. structures such as the prostatic plexus, the intrapelvic fascia, the pubic prostatic ligament, and the detrusor apron in the Retzius space are closely associated with Urinary control and erectile function are closely related (18). The visceral endopelvic fascia, a structure covering the anterior surface of the prostate, fuses with the anterior fibromuscular stroma in the midline to form a pouch-like complex that envelops the prostate and urethra (19, 20). The Pubic prostatic ligament complex is also closely associated with urinary control and erectile function (21). The puboprostatic ligament anchors the bladder, prostate and membranous urethra at the pubic symphysis; The the fascial tendinous arch of pelvis is a fused portion of the mural and visceral components of the intrapelvic fascia that extends from the puboprostatic ligament to the sciatic spine; the puboperinealis muscle is a paired muscle of pubic bone origin located at the prostate-urethral junction and terminating most at the perineal body, which acts as a “hammock “ and supports the urethra posteriorly and is responsible for the cessation of urination (22, 23). In addition, studies have confirmed that the apron of the detrusor muscle contributes to the attachment of the bladder to the pelvis and facilitates the stability of the bladder neck (21). Furthermore, the bladder sphincter and urethral sphincter are muscles recognized as playing a crucial role in the male voiding mechanism (24). The contraction of the bladder sphincter pulls the urethra while moving the urethral sphincter in a dorsal and caudal direction (25, 26). This contraction combined with the upward lifting contraction of the pubococcygeal perineal muscle creates a dual sliding mechanism to close the urethra (26).

Regarding the mechanism by which preservation of the Retzius space facilitates early recovery of urinary control. A recent study by Chang et al. confirmed that patients who retained the Retzius space RARP had a low postoperative bladder mobility, most of the structures adjacent to the membranous urethra were intact, and the bladder neck presented a more anatomical position after surgery (27). Kadono et al. used dynamic MRI to reveal the bladder wall during the abdominal pressure before urethral closure mechanism study, keep the Retzius space after bladder wall is higher than the standard RARP at a fixed location before, because in the process of abdominal pressure fixed before the abdominal wall and the surface of the bladder, bladder former surface as protection, therefore, making the pelvic other organs to the bottom of the slide. The rectum moves forward under the action of abdominal pressure, resulting in the closure of the membranous urethra under pressure from the rear (28, 29). Although Kadono studied patients who retained the Retzius space through the posterior approach, the modified anterior approach preserves the midline umbilical ureteric ligament on the same principle, thus bringing the bladder closer to its anatomical location. This is advantageous for urethral closure during abdominal pressure and is less likely to cause stress incontinence than the standard anterior approach RARP.

The study also has several limitations. 1) Although it was a study of propensity to match scores, which minimized baseline differences between the two groups of data, the results of this analysis were limited by residual selection bias, attrition bias, and possible confusion due to the lack of prospective randomization associated defects between the SA-RARP and MA-RARP groups. 2) Although the distribution of nerve sparing techniques among the groups may be a meaningful confounder, it was not summarized in our study because the anatomical methods of neurovascular bundles differ between surgical techniques, making it impossible to make a pure comparison. 3) Our findings are limited to a single center and a single surgeon’s experience, and a well-designed multicenter randomized controlled trial is needed to determine the stronger advantages of a modified anterior approach to preserve the retzius space RARP.

## 5 Conclusions

In conclusion, the modified anterior approach preserves the Retzius space for robot-assisted laparoscopic radical prostatic intrafascial resection with less surgical steps, less difficulty, and a short learning curve. Compared with the standard anterior approach, the support and suspension structure of the posterior pubic space can be preserved. Compared with the posterior approach, it has a clearer surgical field and is easier to locate anatomical landmarks. This technique is safe and feasible, and it can better preserve the anatomical structure around the urethra, thus better preserving urinary control and sexual function. Since this procedure is an intrafascial resection technique, it is suitable for patients with early localized prostate and small prostate size. At the same time, we are looking forward to more large sample and multi-center clinical data to further verify the efficacy and safety of this technology.

## Data availability statement

The raw data supporting the conclusions of this article will be made available by the authors, without undue reservation.

## Author contributions

HL is the first author. RC, YZ, KW are co-corresponding authors. CY and ZL also contributed to this article. All authors contributed to the article and approved the submitted version.
